# The Action and Administration of Chloroform

**Published:** 1896-06

**Authors:** Luther B. Grandy

**Affiliations:** Atlanta, Ga.


### THE ACTION AND ADMINISTRATION OF CHLOROFORM.*

By LUTHER B. GRANDY, M.D.,

Atlanta, Ga.

I make no apology for presenting a paper with this title to the consideration of this Association. The anesthetic, like the poor, is always with us. Other subjects, medical and surgical, may arise from time to time to receive the predominating attention and interest of the hour, but the ever-present and all-important matter of the anesthetic runs its even and constant course, interrupted only now and then by the invention of a new and useless inhaler, or a so-called improved method of giving the anesthetic.

A great deal of time and work and mental energy have been unnecessarily expended upon both sides of the imaginary case of *Chloroform vs. Ether*, and the merits and attractions of the one have been presented as fully and carefully as the disadvantages and dangers of the other. The discussion is quite superfluous except so far as it has served to point out the special conditions suitable and unsuitable for each anesthetic. Chloroform and ether are in a large measure the complements of each other. They have been used side by side since anesthesia was discovered, and they occupy about the same relative positions now that they did forty years ago. Each has its advantages and its proper field of usefulness, and we could ill afford to do without either.

While it is the object of this paper to present some thoughts upon the action and administration of chloroform, I wish to say right now that for the general purposes of an anesthetic in the hands of all persons who may be called upon to administer it, ether is the preferable agent from the standpoint of safety alone. The

---

* Read by title before the Georgia Medical Association, Augusta, April 16, 1896.

matter of safety outweighs every other consideration. Ether is the anesthetic of election. Chloroform is the anesthetic of necessity, which should be employed only in the presence of a special indication and by some one trained to use it. The death rate from chloroform (from recorded cases) may be put down as one in three thousand cases; from ether, one in ten thousand. Under chloroform the margin between surgical narcosis and a toxic effect is narrow and often precarious; under ether it is broad and comparatively safe. In the South the general use of chloroform for all purposes of anesthesia is much too common, and I am sure that there have been many deaths from it that have never seen the light.

But chloroform cannot and should not be dispensed with. Any one may be called upon at one time or another to administer it, and it is vitally important that it be administered properly.

First, as to the action of chloroform. In late years, thanks largely to the investigations of the Hyderabad Commissions, and to counter investigations conducted by other observers, we have been furnished with an abundance of literature and information upon the phenomena of chloroform anesthesia, and while their results have not been altogether harmonious, they have helped to advance the truth and their influence will be for good. The first chloroform commission appointed by the Nizam of Hyderabad in 1888, after two hundred experiments upon one hundred and forty dogs, announced the following results: (1) Respiration ceased from one-half minute to six minutes before the pulse; (2) the heart continued to beat from three to thirteen minutes after the pulse stopped; (3) artificial respiration restored the animal in every case when commenced within fifty-two seconds after normal respiration had ceased; (4) artificial respiration commenced after the pulse could not be felt was successful in twenty-nine cases and failed in seventeen cases; (5) artificial respiration was unsuccessful in every case where it was commenced after the heart had ceased beating. The second commission (1890) verified the conclusions of the first and added: (1) That death from chloroform is never due to cardiac failure, and (2) that ether is quite as dangerous as chloroform if enough is used to produce true anesthesia. The third commission (1893), consisting of Doctors Hare and Thornton of Philadelphia, agreed in the main with the other two. But these gentlemen made the important concession that while respiratory failure, as a rule, precedes cardiac failure in a healthy animal, the reverse may be the case in a human subject whose cardiac strength and vitality is already lowered by disease. The remarkable statement that ether is as dangerous as chloroform is refuted by clinical experience and figures, the world over, ever since anesthesia has been employed.

Now it must be borne in mind that the above conclusions were based solely upon experiments on healthy dogs. How far we are justified in applying them to the human subject, and to what extent they will be found to conform to clinical experience are open questions, and the most important to be determined after all. Indeed, the final settlement of this, as well as other questions upon which animal experimentation is brought to bear, must be made on the operating table or at the bedside. A human being is neither a dog, a white rabbit, nor a guinea pig, and no amount of laboratory deductions and ratiocinations can take the place of intelligent observations and conclusions based upon actual clinical experience with diseased man. I am afraid that we may be led into a fatal error if we allow ourselves to be guided too much by the Hyderabad conclusions in administering chloroform to man. The warning of Semmola, a few years ago, with reference to laboratory therapeutics in general is as applicable here as elsewhere: "Every so-called scientific construction of the evolution of this or that disease which is based on the investigations of the laboratory will remain a hypothetical structure built upon at least three-fourths of falsehood; and hence form a dangerous guide for the physician who would regard it as the true key to treatment. We should lull ourselves with no illusions in this respect, and I hope that rising physicians will satisfy themselves on this score, that they may not later become murderers of their kind in the name of progress."

Clinical evidence is abundant and conclusive that people have died under chloroform from a sudden failure of the heart and circulation, whether this failure be due to direct effect on the heart muscle or to reflex inhibitory influences through the pneumogastric. Our text-books on materia medica have taught for forty years that chloroform undoubtedly exerts a steady and powerful depressing effect on the heart, and that syncope in many cases is very probably the result of its direct influence upon the heart muscle.* Dr. H. C. Wood says (Dennis's *System of Surgery*) : " Whatever the results of experimentation with anesthetics upon the lower animals may be, it seems to me absurd for any one to claim that in man chloroform does not frequently produce death by an action upon the heart. In numerous cases the heart's action has been well tested with full knowledge on the part of the observer that leaders in the profession have declared that chloroform never kills by its action upon the heart, and yet in nearly four cases out of five it has been noted that death came through the heart. If clinical observations upon the simplest questions be of any value whatever, the conclusion must be that in the large majority of cases chloroform death has been due to cardiac arrest." Doctor Wood thinks that physiologists have completely demonstrated that " chloroform is a direct depressant and paralyzant to the heart muscle or its contained ganglia ; and that the early fall of blood-pressure which occurs in chloroformization is in great part, if not altogether, due to this direct depression upon the heart." MacWilliam has shown (*British Medical Journal,* October 11, 1890,) that dilatation and weakening of the heart occur during chloroform anesthesia, often early in the administration; that it often occurs rapidly, and that cardiac failure is due to this sudden dilatation and enfeeblement of the organ. He believes that while respiration usually ceases before heart failure, the latter may, and does sometimes, take place a considerable time before the former. The depressing effect of a distended and enfeebled heart is due directly to the action of chloroform upon the heart mechanism.

If now we appeal to clinical experience and examine the records for deaths under chloroform, we find that cases will usually read something like this:

1. Female, thirty-four years. Operation for fistula in ano. Three drams chloroform given. As fistula was divided patient screamed, face became pale, pupils dilated, heart ceased to beat. Respiration ceased in few minutes. *Autopsy:* Heart collapsed, pale, covered with fat, its walls were thin, and showed fatty degeneration.

*Therapeutics. Its Principles and Practice. H. C. Wood.

Or like this:

2. Male, middle-aged. Amputation of crushed thumb. After few inhalations, patient died. *Autopsy:* Heart fatty, valves diseased.

Or like this:

3. Male. Removal of diseased bone from leg. Heart and lungs examined and pronounced healthy. Only small quantity chloroform given. Patient died three minutes after beginning inhalation. *Autopsy:* Negative.

Or like this:

4. Male, fifty-one. Dressing fracture of the leg. Half dram chloroform given and in two minutes another half dram. Patient became quiet with regular pulse and respiration. After dressing was applied heart stopped, pupils dilated, respiration continuing; latter soon ceased. *Autopsy:* Atheroma of coronary arteries, heart slightly fatty, right side of heart filled with dark blood, ventricles thin.

Dr. H. M. Lyman, in his excellent work on *Anesthesia and Anesthetics*, from which the above cases have been taken, collected four hundred and ten cases of death from chloroform up to 1880. In many of them, where it is not expressly stated that the heart was the first to fail, the circumstances of the death make it reasonable to assume that this was the case; primary cardiac failure is the only rational interpretation that can be placed upon the fatal result. An examination of recorded cases of death from chloroform warrants the statement that more than one-half occur before the period of full insensibility is reached, and in point of time within the first five or six minutes after inhalation is begun, and before a paralysis of the respiratory centers is to be expected. On autopsy, the hearts of persons so dying often show a weakening and thinning of the walls, a fatty deposit on the heart, or a true fatty degeneration of heart tissues or valvular lesions, or some of these combined. Given such a diseased heart (and you cannot tell always when a heart is diseased), acted upon by a powerful cardiac depressant and paralyzant in the hands, possibly, of one unskilled in its use, and a bad result is not to be wondered at.

Now, no one has ever denied, so far as I know, that primary respiratory failure may happen and does happen. I believe that either heart or respiration may fail first, or they may fail together, depending upon causes and conditions which we do not at present entirely understand. Out of three hundred and eighty-four deaths analyzed and tabulated some years ago by a commission acting for the London *Lancet*, in two hundred and twenty-seven the pulse is said to have ceased entirely before respiration; seventy-seven times they ceased simultaneously, and eighty times the respiration stopped before the heart.

However interesting it may be after a patient is dead for one to have noted all the peculiar circumstances attending his untimely taking off, it is infinitely more important for the chloroformist to guard every avenue of disaster and watch for every signal of danger while he is living. I believe that carelessness in the administration of this anesthetic, including the use of it by persons who do not know how to give it, will explain many of the unfortunate results. It is far too common for the operating surgeon to delegate the least experienced of his assistants to give the anesthetic, and this applies to ether as well as chloroform. Americans are more careful of the anesthetic than Europeans, and our death-rate is correspondingly low. In Great Britain alone, between 1880 and 1889 inclusive, there were one hundred and thirty deaths from chloroform. Last year sixty-one deaths from anesthetics occurred in England and the Provinces. Fifty-two of these were due to chloroform. An American surgeon, lately returned from abroad, says: "I can scarcely wonder at the frequent deaths from chloroform which one sees so constantly reported in the medical journals abroad. In six weeks I saw six patients within an ace of dying. The old rule that chloroform should always be given with plenty of air, seemed to be almost ignored. The chloroform was poured in considerable quantities on a towel or napkin, which was pressed close down on the patient's face, so that he breathed almost absolutely pure vapor of chloroform. In each of the six instances I speak of the surgeon had to discontinue his operation, and the patients were restored with difficulty by artificial respiration and rhythmic traction of the tongue."

How, then, should chloroform be administered? I wish to say at once that if pure chloroform be used, by a person who understands it, and the patient be reasonably healthy, at least so far as his lungs and heart are concerned, the danger is reduced to a minimum, and the patient is correspondingly safe. The dangerous impurities of chloroform consist of certain hydrocarbons, aldehyde, free chlorine, and certain methyl compounds, and decomposition products from exposure to light and air, such as acetic and formic acids. If in doubt about the quality of the drug, pour a few drops upon a piece of clean, white paper. After evaporation, if the chloroform is pure, there will remain no odor and no stain. Chloroform should be kept in a dark place, in ground-glass-stopped bottles. Five drops of alcohol to the ounce of chloroform will help to preserve it, without interfering with its virtues as an anesthetic.

The patient's health, and the integrity of his heart and lungs especially, should be in such condition, as far as can be ascertained, as to render the inhalation of chloroform reasonably safe. The chief danger, on the side of the heart, is from fatty degeneration and the consequent weakening of the heart muscle. Endocarditis—even with a moderate valvular lesion—need not debar the use of chloroform (ether being contraindicated)), provided the force and strength of the heart muscle are still maintained. The patient's chest should be examined in a quiet manner and his attention diverted, if possible, from the purposes of the examination. The chloroform should be given, a few drops at a time, on an Esmarch mask. With children, a handkerchief may be used with safety. The patient should be told to breathe naturally. The anesthetic should be "pushed" with extreme caution, especially at first. If the patient be violent and inclined to scream and struggle, special care should be taken to prevent a sudden inhalation of strong chloroform vapor, or ether should be substituted for the chloroform.

Rules have been drawn up with much care about watching the respiration, feeling the pulse and noting from time to time the condition of the pupil, as the patient goes into and remains in the state of anesthesia. Each of these indexes has been advocated by different observers as furnishing a sufficient guide to the patient's actual condition, and the president of the first Hyderabad Commission summed up the duties of the anesthetizer in these words:

"Use pure chloroform, watch the respiration and keep it regular." Others tell us to judge of the patient's condition from the pulse or the pupil. Such I believe to be bad advice. Chloroform or ether cannot be properly administered by rule. Be governed by the patient, and not by a rule. Neither of the above indexes should be depended upon to the exclusion of others, and the man who loses a patient from a failure to take advantage of every available means of information as to his patient's condition would certainly be held culpable. The anesthetizer, either with chloroform or ether, should watch respiration, pulse, pupil, and color of the face. With an intelligent observation of these four things, and a ready appreciation of unfavorable symptoms, on the part of an anesthetizer who confines his attention to his own work and lets the operation alone, there should be fewer accidents, and rarely a death from any anesthetic. About three months ago I gave chloroform to a young man from whom a cystic testicle was to be removed by Drs. Nicolson and Westmoreland. The anesthetic was poured on an Esmarch mask, a few drops at a time, and for several minutes the patient took it fairly well. After about fifteen minutes the face turned pale, the pulse was feeble and frequent, the respiration shallow, and the pupils became suddenly and widely dilated. His condition was the most alarming that I ever witnessed, and nothing but the instant recognition of the danger and the immediate resort to strychnine and artificial respiration, etc., saved the patient's life.

The pupil, I believe, is the most reliable single indication, and, as a rule, neither the heart nor the respiration will be materially affected before a distinct and appreciable impression is made upon the pupil. The pupil is controlled by the third-nerve center in the aqueduct of Sylvius. As the patient goes under the anesthetic certain changes may always be noted in the pupil. At first it dilates slightly from the sensory impression on the cerebrum, just as momentary dilatation may be produced by the inhalation of any pungent vapor or by emotion. In the second, or surgical stage, when the cerebrum is paralyzed and no longer appreciates sensory impressions, the pupil is contracted, because the third-nerve center is still active. If now the anesthetic is pushed still further and this center is paralyzed, dilatation of the pupil results. By the time this occurs an embarrassment of the respiration may be noted, and a weakened heart action and a pallor or cyanosis of the face. Then comes the tug of war. But the patient should not be allowed to reach this point if strict attention to business and a careful watch for danger signals can prevent it. A pupil contracted and responsive to light is the limit beyond which it is not only not necessary, but extremely dangerous, to go. It is needless to add that digitalis and strychnine should always be on hand, and at hand in case of accident.

I have several times noted among physicians an unwillingness very much akin to fear to administer chloroform. I have seen some decline to give it, and I have seen others give it who certainly did not understand it, and there are others who will indulge in the wildest talk about the awful dangers of chloroform and express themselves as opposed to its use in almost any circumstances. Such fears and ideas are unreasonable, and if doctors generally were better acquainted with the details of chloroform administration, as they should be, there would be less of such prejudice.

One word in closing about the ACE mixture, and that is a word of warning. Whatever theoretical considerations are responsible for its existence and use break down under the brute force of practical results. The death-rate from this mixture is about the same as that from chloroform. The mixture is usually administered in a towel and paper cone, after the manner of ether, and after a few minutes inhalation the ether is evaporated and the patient is breathing a strong vapor of chloroform in a closed space with an insufficient supply of air. The composition of this mixture lulls the anesthetizer into the false belief that the patient is breathing a vapor with a constant proportion of one part alcohol, two of chloroform, and three of ether. This ratio does not exist in the vapor of this mixture. Similarly with any mixture of chloroform or ether. These drugs when put together do not produce a new chemical compound, as is sometimes claimed by advocates of the ACE mixture, but they remain a simple mixture, and each part evaporates in its own time. If this mixture is to be used at all, it should be administered with all the care of chloroform, because the ether in it is rapidly dissipated, and in the end we practically get a chloroform anesthesia.

I have endeavored in this paper to show that for the general purposes of anesthesia ether should be employed in preference to chloroform; that chloroform, however, is indispensable in its place; that it is the duty of every physician to know how to administer it properly, and that if given carefully by a person duly watchful of his patient's condition the danger from it is reduced to a minimum; that the ACE mixture has no advantage over chloroform or ether, but is more dangerous than either, and that if used at all it should be administered as chloroform.

